# Youths’ Wellbeing Between Future and Uncertainty Across Cultural Contexts: A Focus on Latent Meanings as Mediational Factors

**DOI:** 10.3390/ejihpe15120244

**Published:** 2025-11-29

**Authors:** Massimo Ingrassia, Narine Khachatryan, Simone Rollo, Edita Arakelyan, Tsaghik Mikayelyan, Loredana Benedetto

**Affiliations:** 1 Department of Clinical and Experimental Medicine, University of Messina, 98124 Messina, Italy; loredana.benedetto@unime.it; 2 Department of Personality Psychology, Yerevan State University, Yerevan 0025, Armenia; n_khachatryan@ysu.am (N.K.); edita.araqelyan@ysu.am (E.A.); tsaghik.miqayelyan@ysu.am (T.M.); 3 Department of Translational Biomedicine and Neuroscience, University of Bari Aldo Moro, 70121 Bari, Italy

**Keywords:** wellbeing, distress, future time perspective, intolerance of uncertainty, worry for war, worry for climate change, latent meanings, youth

## Abstract

Factors like future time perspective, cultural belongings, and semiotic resources (i.e., individuals’ meanings to interpret the world), as well as worrying phenomena (climate change and armed conflicts), can harm wellbeing and increase personal distress. The study, comparing Armenian and Italian contexts, explores whether youths’ wellbeing and psychological distress are explained by openness to time perspective, anxiety about uncertainty, and worry regarding climate change and war, as a function of the individual semiotic resources (mapped by *Views of Context*). Participants were 202 Armenian and 271 Italian young adults (*M*_age_ = 21.23, *SD*_age_ = 3.35). A Multiple Correspondence Analysis (MCA) applied to *Views of Context* extracted two dimensions of sense; a second-order MCA aggregated the extracted meanings into three clusters named *Orientation towards self-care* (CL_1_), *Social and personal commitment* (CL_2_), and *Absolute devaluation and social detachment* (CL_3_). Clusters and/or nationality significantly differentiated measures of worry for war and climate change, future time perspective, intolerance of uncertainty, and wellbeing, but not the distress scores, by 3 × 2 ANOVAs. Linear regressions showed future time perspective and intolerance of uncertainty as positive and negative predictors of wellbeing respectively, with a significant *Views-of-Context* dimension, inversely affecting distress scores. Study highlights youths’ latent meanings influence wellbeing and distress, serving as a “starting point” for health promotion interventions sensitive to cultural differences.

## 1. Introduction

There is a global consensus about a decline in wellbeing and mental health among young adults over the past two decades (e.g., Blanchflower, 2025; Twenge & Blanchflower, 2025). This trend appears especially prominent in Western Europe and English-speaking advanced economies, but it remains controversial for those living in Eastern Europe and Central Asia nations (Blanchflower & Bryson, 2025).

In Italy, the latest report from Istat (Istituto Nazionale di Statistica) on fair and sustainable wellbeing states that the mental health indicator (68.7) in 2023 “remains stable even compared to 2019 (68.4), but, in the face of this relative stability, starting from 2020 a worrying decline in psychological wellbeing has been observed, especially among the youngest, particularly girls” (Istat, 2024, p. 37). Similarly, Samokhvalova et al. (2022) reported a decrease in the overall level of psychological wellbeing among Armenian and Russian university students.

Young adulthood (ages 18–34, according to the United States Census Bureau, e.g., Anderson et al., 2023) is a life stage marked by important transitions that challenge an individual’s wellbeing: completing education, entering the workforce, and starting a family are some of these challenges (Rod et al., 2025). Additionally, identity experiments and explorations are very likely, especially during the 18–28 age range (Arnett, 2000). It is a period of significant project planning; thus, a future time perspective becomes relevant for mentally projecting oneself into conditions not yet attained (e.g., graduated, professional, married, parent, and so on).

Many authors emphasise the importance of time perspective in human development (for a historical and theoretical review, see Stolarski et al., 2018), affecting youths’ motivation, personal growth, and wellbeing across various life areas (Kooij et al., 2018). An optimistic and expansive outlook on the future is associated with better psychological wellbeing (Burzynska & Stolarski, 2020; Drake et al., 2008; Zambianchi, 2019), increased openness towards external relationships (Lang & Carstensen, 2002; Zambianchi & Ricci Bitti, 2012), and a strong link with individual motivation to plan and take action (see e.g., Stolarski & Matthews, 2016; Zimbardo & Boyd, 1999).

In young adults, a mental projection into the future may be compromised by various personal characteristics and societal phenomena. A noteworthy personal characteristic is the intolerance for uncertainty, which encompasses both prospective anxiety—defined as the tendency of individuals to actively seek information to reduce uncertainty—and inhibitory anxiety, involving paralysis or avoidance responses when confronted with uncertainty (Carleton et al., 2007). Several societal phenomena can amplify the perception of uncertainty regarding one’s own future and the overall progression of humanity. As Volker Türk articulated in his opening address to the 57th Human Rights Council in Geneva, «It seems to me we are at a fork in the road. We can either continue on our current path—a treacherous ‘new normal’—and sleepwalk into a dystopian future. Or we can wake up and turn things around for the better, for humanity and the planet. […] The ‘new normal’ cannot be endless, vicious military escalation and increasingly horrifying, technologically “advanced” methods of warfare, control, and repression. The ‘new normal’ cannot be continued in indifference to deepening inequalities within and between States» (Türk, 2024): in reality, numerous events can be identified that evoke uncertainty about the future.

Two societal contingencies that may significantly challenge perceptions of the personal future are armed conflicts, particularly for individuals residing in directly affected countries, and the severe local repercussions of the climate crisis, such as flooding and famine. In Armenia, Vermishyan et al. (2023) found that the majority of young Armenians (aged 14–29) were preoccupied with war and the climate crisis. A significant portion of Armenian youth believed, during May and June 2022, that climate change represents a global threat, experiencing emotions such as anger, helplessness, indifference, and fear; few expressed hope and confidence (see also Nartova-Bochaver et al., 2022). Furthermore, they anticipated the resumption of the Karabakh war within the next five years (which actually occurred on 19 September 2023, when Azerbaijan launched a large-scale military offensive against Nagorno-Karabakh, resulting in the forced flight and displacement of the local Armenian population [cf. *2023 Azerbaijani offensive in Nagorno-Karabakh*, 2025]). Despite these concerns, many young individuals remained optimistic regarding the future, believing that their families’ living conditions would improve within five years (Vermishyan et al., 2023, p. 8). In Italy, Barchielli et al. (2022) emphasised that younger adults constitute the demographic group most concerned about war, followed by concerns related to climate change and natural resource depletion. Italian youth demonstrate a greater tendency to plan for the future than older adults; additionally, their anxieties are associated with psychological distress, anxiety, and depression. Similarly, Regnoli et al. (2024) confirmed that the Russian-Ukrainian conflict exerts a negative psychological impact even on communities, such as Italian young adults, that are not directly affected by war. Moreover, the fear of war was mediated by intolerance of uncertainty, which increased levels of anxiety, depression, and distress. Consequently, it appears pertinent to investigate the impact of these two contemporary challenging phenomena on the mental health of youth, as evidenced by studies such as Clayton (2020) and Scharbert et al. (2024).

Mental health constitutes «a state of mental wellbeing that enables people to cope with the stresses of life, realise their abilities, learn well and work well, and contribute to their community. It is an integral component of health and wellbeing that underpins our individual and collective abilities to make decisions, build relationships, and shape the world we live in. Mental health is a basic human right. And it is crucial to personal, community, and socio-economic development» (WHO, 2022, p. 8). Regrettably, «the diversity of cultural meanings given to mental health and wellbeing terms across countries, communities, and age ranges adds an extra layer of complexity to measuring mental wellbeing internationally in a standardized manner» (European Public Health Alliance, 2025, p. 39). Numerous indicators have been suggested for assessing the levels of mental health and wellbeing at both population and individual levels (e.g., see Peitz et al., 2021): some focusing on identifying mental distress, while others aim to highlight positive aspects of wellbeing.

Positive (as hedonic and eudaimonic wellbeing indexes) and negative (as anxiety, depression, and stress indexes) characteristics may be considered as two complementary (correlated) facets of the same “mental health” concept; they are complementary but not symmetrical: if distress levels are elevated, wellbeing is likely to be low, and vice versa. However, a low level of wellbeing does not necessarily imply important distress. As outlined by the Dual Continua Model (Keyes, 2005; see also Westerhof & Keyes, 2010), mental health and mental illness are not merely opposite ends of a single continuum; rather, they are distinct yet correlated dimensions.

In this study, we represent youths’ wellbeing using two widely adopted and culturally validated questionnaires: the *Mental Health Continuum-Short Form* (MHC-SF; Keyes, 2018), which assesses hedonic and eudaimonic wellbeing, and the *Depression Anxiety Stress Scale-21* (DASS-21; Lovibond & Lovibond, 1995), which measures overall distress. The former provides a positive indicator of mental health, while the latter indicates negative aspects of mental health.

Furthermore, cultural belonging and semiotic resources—namely, the meanings individuals adopt to interpret the world—may influence how individuals cope with challenging life experiences. From a semiotic and cultural perspective, individuals construct the meanings of their life experiences within the contexts to which they belong (Salvatore et al., 2019). Consequently, meanings guide their actions and thoughts. This sensemaking process—developing through feeling, thinking, and decision-making—is directed by generalised meanings, defined as “specific concepts and opinions regarding facts and objects of the social and physical world” (Ciavolino et al., 2017, p. 600). For instance, meanings oriented toward social commitment (such as caring for others and the planet) may contribute to intensifying symptoms of distress, as individuals perceive themselves as helpless in the face of the climate crisis, which is, in turn, attributed to short-sighted energy policies enacted by governments. Numerous studies have established the association between meanings and distress. For example, research has demonstrated that particular representations of one’s experience within everyday contexts distinguish pathological gamblers from control group individuals (Venuleo et al., 2015a), as well as from individuals with other addictive behaviour-related disorders (Venuleo et al., 2015b, 2016). Additionally, among adolescents, the perception of risk is directly influenced by the ways in which individuals interpret and make sense of their experiences in the world (Venuleo et al., 2019). Ultimately, meanings result from an interaction between individual and collective subjectivities within the cultural context of belonging. Considering this, it is not war in itself that is frightening, nor climate change; it is the meaning that these specific aspects take on in light of specific semiotic configurations. An example is the latest global health crisis linked to COVID-19. During the pandemic, Internet use has played an important role in the daily lives of many people. Venuleo et al. (2022) demonstrated how being online could acquire very different meanings depending on the context and the individual’s subjectivity. In their research on young Italian adults, the authors identified two primary dimensions in the way the Internet was experienced: firstly, as a tool to maintain continuity in daily routines and support activities in a context of disruption (i.e., the pandemic) compared to normal daily activities; and secondly, as a refuge to escape the distress caused by the health emergency. Specifically, they found that perceiving the Internet as a resource is associated with greater psychological wellbeing, while viewing it as a refuge is correlated with increased distress. This finding implies that it is not merely the use of the Internet that determines wellbeing or distress, but rather the meaning ascribed to that use within the individual’s life. Consequently, particular configurations of meanings might mediate or moderate the potential relationship between wellbeing and personal or social factors of uncertainty and distress.

Based on these theoretical premises, this study primarily aims to examine wellbeing and distress among Armenian and Italian youth. Using Keyes’ model, the prevalence of mental health statuses—flourishing, moderate, and languishing—will be compared between the two national groups. Additionally, the study will explore how personal and environmental factors might influence young people’s wellbeing. Lastly, the impact of generalised meanings will be assessed.

The research questions and hypotheses of the study are:

Q1: Are there differences in wellbeing (measured by MHC-SF) and general distress (measured by DASS-21) between Armenian and Italian youth?

**H1.**  *As reported in the Introduction, current studies have highlighted a decline in wellbeing of both young Armenians (Samokhvalova et al., 2022) and young Italians (Istat, 2024). However, no previous studies have compared the wellbeing of young people between these two populations according to Keyes’ Dual Continua Model. Whereas, regarding general distress, we expect higher levels among Armenian youth, who have closer experience of armed conflict (cf. Movsisyan et al., 2022), compared to Italians.*

Q2: Does the intolerance of uncertainty negatively affect wellbeing?

**H2.**  *Participants experiencing greater uncertainty will exhibit more pronounced negative emotional symptoms and diminished levels of wellbeing.*

Q3: Is a future orientation—positively defined as an expectation for upcoming opportunities, plans, and future objectives (Carstensen & Lang, 1996)—linked with higher levels of wellbeing?

**H3.**  *The perception of increased opportunities and planning prospects in the future is associated with enhanced wellbeing.*

Q4: Are societal sources of worry, such as war and the climate crisis, correlated with wellbeing and general distress of Armenian and Italian youth?

**H4.**  *Higher worry levels for war and/or climate change are associated with reduced well-being and higher general distress.*

Q5: Do latent dimensions of sense (i.e., general meanings) mediate the relationships between levels of wellbeing and/or general distress and other measures (i.e., intolerance of uncertainty, future-oriented time perspective, worries about war and/or climate crisis)?

**H5.**  *The hypothesis is that specific semiotic configurations mediate the effect of social sources of worry (war and/or climate change) on the levels of wellbeing and general distress.*

## 2. Materials and Methods

### 2.1. Participants

A total of 473 youth participants (*M*_age_ = 21.2; *SD* = 3.3; 72.9% females) were recruited from Armenia (*n* = 202; 74.3% female; *M*_age_ = 21.2, *SD* = 2.32) and Italy (*n* = 271; 71.1% female; *M*_age_ = 21.3, *SD* = 3.94). Appendix A.1 “Participants” provides more detailed information on recruitment and sample characteristics.

### 2.2. Measures and Data Collection Procedure

All measures were combined into a comprehensive questionnaire including: wellbeing (*Mental Health Continuum-Short Form* [MHC-SF; Keyes, 2018]), general distress (*Depression Anxiety Stress Scale-21* [DASS-21; Lovibond & Lovibond, 1995]), intolerance of uncertainty (*Intolerance of Uncertainty Scale-Revised* [IUS-R; Bottesi et al., 2019; Carleton et al., 2007]), future time perspective (*Future Time Perspective Scale* [FTP; Carstensen & Lang, 1996; Lang & Carstensen, 2002]), worry for climate change (*Climate Change Worry Scale* [CCWS; Stewart, 2021]) and war (*War Experience Worry Scale* [WEWS; Rollo et al., 2023]), lastly view of the context (*Views of Context* [Ciavolino et al., 2017]). After obtaining informed consent, the questionnaire was made available online via a link or QR code in Google Forms. The participant’s demographic characteristics (i.e., gender, age, social status, and educational attainment) were also collected. Two versions of the questionnaire in Armenian and Italian were developed. Data were collected from May to October 2024 in Armenia, and from September 2023 to July 2024 in Italy. Appendix A.2 “Measures” details the characteristics of all administered instruments.

### 2.3. Statistical Analysis

An initial analysis aimed to identify the principal interpretative keys (i.e., the Latent Dimensions of Meanings, or cultural meanings) adopted by participants to interpret their experiences in social contexts. The responses from the total sample (N = 473) to the *Views of Context* were examined using Multiple Correspondence Analysis (MCA; Duncombe, 1985; see Appendix A.3 “Multiple Correspondence Analysis”). As an additional step, concerning the factorial dimension extracted, groups of meanings (above “semiotic clusters”) based on similarities in the relationships between variables and response modalities were identified.

The other analyses were aimed at:

(1) through 3 (Semiotic cluster) × 2 (Group) ANOVAs and Chi-square test estimating the differences (Q1) in MHC-SF (including the prevalences of wellbeing diagnoses) and DASS-21 between Armenian and Italian participants. Additionally, as a function of national groups and semiotic clusters, differences were tested in all other measures. A further analysis examines the distribution of responses within the two populations regarding the items of the two self-report instruments used to identify worries about climate change and war.
(2) Regression analyses were performed to explore the impact of (Q2) IUS-R, (Q3) FTP, (Q4) CCWS, and WEWS on wellbeing and distress.
(3) Finally, mediation models were computed to assess (Q5) how meanings (i.e., Latent Dimensions of Meanings by *Views of Context* [VoC]) mediate the relations between worry about climate change and war, and levels of wellbeing and general distress.

## 3. Results

### 3.1. Detection of Cultural Meanings

Two principal dimensions of meanings were extracted. The first has been interpreted as “Levels of action in the social context” due to its contrast between two polarities: “Commitment to oneself and others” (VoC_1_^NEG^) and “General disengagement” (VoC_1_^POS^). The second dimension of sense has been interpreted as “Relationship with the social context” because it contrasts two polarities: “Detachment and disillusionment” (VoC_2_^NEG^) and “Impotence and loneliness” (VoC_2_^POS^). Based on these two dimensions, three clusters of meanings were extracted: “Orientation towards self-care” (CL_1_), “Social and personal commitment” (CL_2_) and “Absolute devaluation and social detachment” (CL_3_). Appendix A.4 “Dimensions of the meanings” reports a more detailed description of the dimensions of meanings and clusters.

### 3.2. Cluster of Meanings and Cross-Cultural Differences (Q1)

Analyses of variance (ANOVAs) were conducted to investigate differences among clusters of meanings and nationalities concerning wellbeing (MHC-SF), general distress (DASS-21), intolerance of uncertainty (IUS-R), future time perspective (FTP), and worries for climate change (CCWS) and war (WEWS). The findings indicated significant distinctions between meaning clusters for intolerance to uncertainty (IUS-R; F_2, 467_ = 7.284; *p* < 0.01), with no considerable differences observed concerning nationality. Specifically, people in the “Social and personal commitment” cluster (CL_2_) exhibited higher scores on the IUS-R for both Italian (Mean = 36.8) and Armenian (Mean = 34.6) participants. Notable variations were also identified in future time perspective regarding both meaning clusters (FTP; F_2, 467_ = 4. 292; *p* < 0.05) and nationality (FTP; F_1, 467_ = 10. 780; *p* < 0.01). In this context, CL_2_ demonstrated higher levels of FTP among both Italian (Mean = 4.6) and Armenian (Mean = 5.1) individuals, with Armenians displaying generally greater future perspective. Concerning climate change, differences were detected among meaning clusters (CCWS; F_2, 467_ = 6.543; *p* < 0.01) with higher scores on the “Social and personal commitment” cluster (CL_2_), and among nationalities (CCWS; F_1, 467_ = 10.790; *p* < 0.001). Italian participants in CL_2_ exhibited greater climate worry (Mean = 26.9) compared to Armenian participants (Mean = 21.6). Worry about war experiences revealed significant differences across both meaning clusters (WEWS; F_2, 467_ = 21.036; *p* < 0.001) and nationalities (WEWS; F_1, 467_ = 18.791; *p* < 0.001). Notably, Armenian participants belonging to the “Social and personal commitment” cluster (CL_2_) expressed greater concern (Mean = 36.3) relative to Italian participants (Mean = 29.8). No differences were identified among meaning clusters concerning wellbeing and general distress; however, a significant difference emerged for nationality in relation to wellbeing (MHC-SF; F_1, 467_ = 5.327; *p* < 0.05), with higher levels observed among Italian participants (Mean = 36.0) compared to Armenian participants (Mean = 32.4). Additionally, in relation to the diagnostic prevalences for wellbeing (i.e., flourishing, moderate, and languishing, as defined by Keyes, 2018), a significant association was found concerning the meaning cluster (χ^2^ = 10.462; df = 2; *p* < 0.05), with languishing primarily characterised by CL_2_. There were no significant interaction effects observed between meaning clusters and nationality across these measures (Table 1 and Table 2). Comparing the distributions of responses (see Appendix A.5, Table A7 and Table A8) within the two populations for items related to worries about war and climate change, we observe that, for the latter, response frequencies tend to be uniform across the sample and are biased towards the “never/rarely” response pattern. In terms of frequency, this indicates that people did not express any worry. However, response frequencies are more varied for worries about war. The highest percentages are for the “often/always” response patterns. Italians respond that they are often worried about the items “I tend to get worried when I hear about war, even when the effects of war may be far away” (44.2%) and “I believe that the increase in severe discord events may be the result of war” (48.3%). Armenians, on the other hand, respond “often/always” to the items “I worry about how the effects of war may affect the lives of people I care about” (73.2%) and “I realised that I was worried about war” (58.9%).

### 3.3. Determinants of Wellbeing and Distress (Q2, Q3 and Q4)

The linear regression model concerning wellbeing (MHC-SF) indicates a statistically significant influence of uncertainty intolerance (IUS-R; t = −3.940; *p* < 0.001), future time perspective (FTP; t = 7.400; *p* < 0.001), the first Latent Dimension of Meanings (“Levels of action in the social context” [VoC_1_]; t = −2.610; *p* < 0.01), and worry related to war experience (WEWS; t = −3.040; *p* < 0.01). No significant effect was observed for climate change worry (CCWS) and the second Latent Dimension of Meanings (“Relationship with the social context” [VoC_2_]).

Instead, the linear regression model pertaining to general distress (DASS-21) reveals significant effects of uncertainty intolerance (IUS-R; t = 11.727; *p* < 0.001), future time perspective (FTP; t = −6.415; *p* < 0.001), and war experience worry (WEWS; t = 2.876; *p* < 0.01), but no significant effects were identified for either dimension of sense (VoC_1_ and VoC_2_) nor for climate change worry (CCWS). Table 3 and Table 4 present the metrics for the linear regression models.

### 3.4. Mediational Role of Cultural Meanings (Q5)

**Figure 1:**
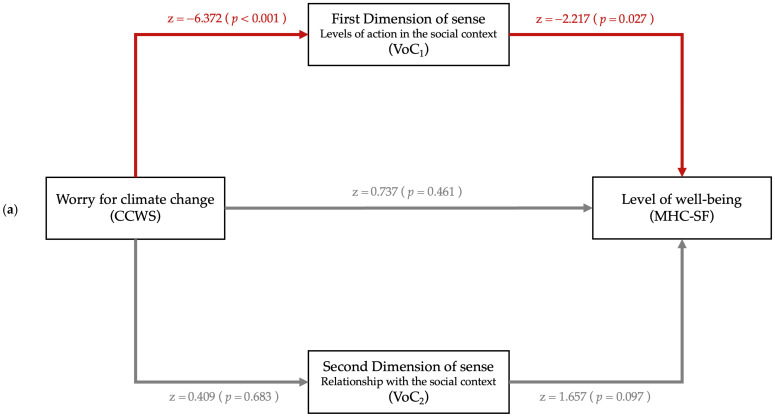

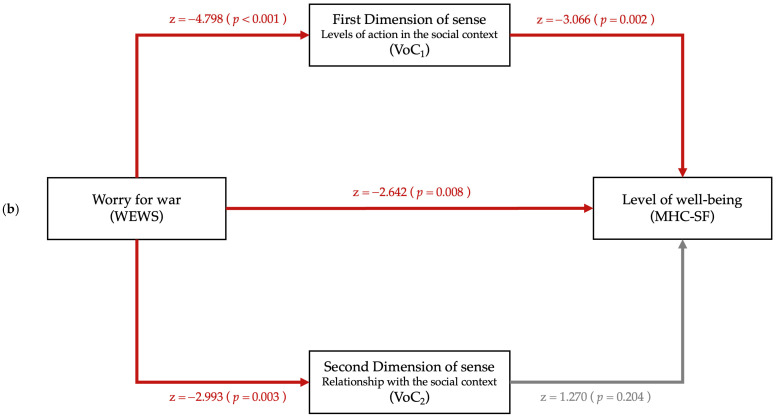
Graph path of mediational analysis specifically for worry about (**a**) climate change (predictor: CCWS; Mediator = VoC; Output Variable = MHC-SF) and (**b**) war (predictor: WEWS; Mediator = VoC; Output Variable = MHC-SF).

The mediation analysis demonstrates the function of the first Latent Dimension of Meanings (“Levels of action in the social context”; VoC_1_) as an intermediary between war experience worry (WEWS), climate change worry (CCWS) and wellbeing (MHC-SF). The study accounted for uncertainty intolerance (IUS-R) and future time perspective as covariates. The metrics pertaining to wellbeing are provided below (see Figure 1, Table 5 and Table 6). No mediational effect was found for general distress (DASS-21; see Appendix A.5, Table A9 and Table A10).

## 4. Discussion

The present study aimed to examine levels of wellbeing and general distress among youth from Armenia and Italy. The main goal was to investigate how wellbeing and general distress vary in relation to the perceived impact of collectively recognised stressors, such as concerns about war and climate change, since research shows that youth are particularly susceptible to and affected by these global crises (Lau et al., 2024). These relationships were also analysed by considering the role of certain psychosocial traits—particularly intolerance of uncertainty and perspective about the future—and semiotic-cultural dimensions, which relate to the meanings underlying human action and cognition.

The first research question focused on potential differences in wellbeing and general distress across different national contexts (Q1). We found differences in wellbeing between Armenian and Italian participants. From a categorical point of view (according to Keyes’ diagnostic model of mental health; Keyes, 2018), flourishing people are more common among Italians (20.3%) than Armenians (15.3%): conversely, languishing people are more common among Armenians (17.8%) than Italians (15.5%), but these differences are not statistically significant. These results are in line with flourishing prevalences observed in 2011 in Europe and referred to nations of Eastern (as Armenia) and Southern/Western continent (as Italy; Huppert & So, 2013). From a dimensional point of view, the Italian participants reported significantly higher wellbeing measures than Armenians. This discrepancy may be related to cultural and historical factors unique to each country. Armenia and Italy have recently faced similar humanitarian crises connected to the COVID-19 pandemic, but only Armenia had to face ongoing conflicts (the 2020 Second Nagorno-Karabakh War) that deeply impacted its social and mental health (Harutyunyan et al., 2021; Kazaryan et al., 2021; Markosian et al., 2021). Furthermore, in a range of 0–70 MHC-SF points, neither the Armenian average score (32.4) nor the Italian average score (36.0) appears to be an indicator of an absolutely high level of wellbeing. Hence, wellbeing should be understood in the context of these historical events and other personal and contextual factors detected by the study. Interestingly, we found not significant differences between Armenian and Italian samples in general distress levels, assessed as symptoms of anxiety, depression, and stress (DASS-21). This may be because the samples were recruited from the general population, not from a clinical group, implying that such symptoms are present but not severe enough to cause distress. As a support of this, a recent study (Koummati et al., 2025) indicates that only a small percentage of participants from a general population experienced mild psychiatric symptoms without meeting criteria for mental disorders. Additionally, the use of a self-report scale, which is not a diagnostic instrument, may have contributed to the absence of variation (Lee et al., 2019).

These results, highlighting a significant difference in wellbeing but not in general distress between the two national contexts, could be understood within a broader framework of historical and social experiences, but also in relation to everyday and personal factors. In particular, while it is known that stressful circumstances in daily life can influence levels of wellbeing and general distress (DeLongis et al., 1988), certain psychosocial traits seem to predispose individuals to varying levels of mental and social health. This perspective also informs the further research questions of the current study: we asked whether the intolerance of uncertainty (Q2) and the inability to project oneself into the future, in terms of future time perspective (Q3), can influence wellbeing and general distress.

Our results indicate that intolerance of uncertainty predicts lower wellbeing and higher general distress. This aligns with studies that consistently show a significant link between intolerance to uncertainty and general distress (Lally & Cantillon 2014; Şentürk & Bakır, 2021). Conceptually, intolerance of uncertainty is the cognitive and emotional tendency to react with anxiety, worry, and anguish when faced with ambiguous, unpredictable, or unknown situations (Birrell et al., 2011; Carleton et al., 2007). Thus, the inability to tolerate uncertainty fuels rumination, avoidance, and excessive control, which sustain high levels of emotional arousal and distress (Gu et al., 2020; Xu et al., 2024). Another key finding of this study relates to the connection between future perspective and young adults’ health. A broader perspective on the future enhances wellbeing, while a narrower perspective heightens distress, as supported by previous research (cf. Pfund et al., 2022).

However, these findings must be interpreted within the context of individuals’ everyday environments. People are not mere psychological systems isolated from their social contexts; they are embedded within and influence them. Social phenomena that disrupt daily routines and hinder future planning may compromise psychological health. Particularly, we hypothesised that high-impact collective phenomena, such as war and climatic crisis, were linked with reduced wellbeing and higher psychological distress (Q4). Notably, worry about war, but not about climate change (as hypothesised), reduced wellbeing and increased general distress. War likely evokes the “fear of destruction” both among those directly involved and those indirectly affected through the media. While direct experience may associate war with death, it also frightens those indirectly exposed. This raises the question: what about war is frightening? Psychologically, war can represent a “rupture” in daily continuity (Walton et al., 1997; Urbanc, 1998), disrupting the normal flow of past, present, and future in an individual’s consciousness, potentially leading to existential crises and a loss of subjectivity; a dynamic that undermines social structures through violent acts (Adams, 1991; Kuznar, 2025). Experiencing social breakdown caused by war, regardless of political causes, psychologically alters one’s perception of what “tomorrow” holds and erodes the ability to plan ahead. The fear of destruction connects the communities affected geographically by conflict with others who have only a mediated view of war and are not directly affected by it (Regnoli et al., 2024). The shared aspect of destruction appears to be an identity crisis: “What will my tomorrow be?”. The persistent projection of a threatening future gradually depletes emotional and cognitive resources, impairing subjective stability and satisfaction. No direct effects were observed for climate change, despite its importance. This may be due to climate change’s delayed impact and, since distress here relates to quality of life rather than clinical symptoms, its influence may be less immediate. Longitudinal research indicates that worry about climate change predicts increased general distress over time, though it does not reduce overall life satisfaction (McBride et al., 2021). Two further explanations might be possible. A further explanation of the absence of evidence could be confined to the construct being investigated. This study employed a self-report measure to assess worry related to climate change rather than anxiety symptoms specific to the environment; in this context, the current literature on eco-anxiety may likely offer different evidence. Indeed, eco-anxiety, characterised by worry, guilt, and sadness, sensitises individuals to situations concerning ecological values, stimulating cognitive engagement and motivation to address the ecological challenges. Therefore, eco-anxiety is an “eco-emotion” that promotes awareness and engagement, positively contributing to environmental management and agency, as well as individual and collective wellbeing (cf. Kurth & Pihkala, 2022). Furthermore, the two contexts exhibit heterogeneity in responses solely regarding war, whereas no such variation is observed concerning climate change. When analysing the highest percentage of respondents who answered “often or always”, Armenians—unlike Italians—demonstrate greater concern about the psychological, emotional, and physical effects that war could have on themselves and their loved ones. Responses predominantly focused on “never/rarely” for climate change indicate that this phenomenon does not constitute a pressing concern for the surveyed populations.

Finally, in line with the semiotic approach (Salvatore et al., 2019), the last research question was: do meanings serve as mediating factors in the relationship between stress-inducing social events (such as war and climate worry) and levels of psychological health? (Q5). The main semiotic configurations were identified. Specifically, two latent dimensions of meaning that participants adopt to interpret their experiences of the world were detected: the first pertains to the level of investment in social context and the community (i.e., “Commitment to oneself and others” *versus* “General disengagement”); the second concerns the relationship with the context of belonging (i.e., “Detachment and disillusionment” *versus* “Impotence and loneliness”). The interaction of these two dimensions subsequently resulted in three clusters of meaning: “Orientation towards self-care”, “Social and personal commitment”, and finally, “Absolute devaluation and social detachment”. These latter dimensions were also identified as mediators in the relationship between stressful events and wellbeing. In the direct effect, less worry correlates with higher wellbeing. However, there is a mediating effect of meaning in this relation. We identified a mediation only of the first latent dimension of meaning (i.e., “Levels of action in the social context”) between worry about war, as well as climate change, and wellbeing.

The meanings identified in our mediation analysis provide valuable insight into understanding wellbeing, not merely as the absence of illness, but as a broader condition in which psychological and socio-cultural dimensions intersect. Several studies confirm that heightened concern about a stressor diminishes levels of wellbeing. Respondents who report high levels of concern about both war and climate change also show higher scores on the well-being scale. These relationships are mediated by meanings that guide commitment to oneself, others, and the environment. Research has identified a relationship between levels of worry and distress. For example, some studies have established links between increased awareness of climate change and heightened depression, anxiety, stress, environmental anxiety, and even suicidal thoughts (Gianfredi et al., 2024; Temte et al., 2019). Similarly, studies on war reveal correlations between threat perception, depression, and anxiety (Lin & Yen, 2024). The levels of well-being present in our study, however, would seem to be explained by the meanings shared by the participants. Here, the introduction of meanings offers a further perspective on explaining this cause-and-effect relationship. First of all, it is interesting how the meanings associated with the different actions respondents take in their own context lead to varying levels of wellbeing starting from the same external trigger (i.e., war or climate). Specifically, our findings suggest that people who are more concerned about war and climate change tend to share meanings that promote investment in themselves and their communities, resulting in higher wellbeing. This can be understood through participation and collective responsibility (Moscovici, 1981; Mannarini & Fedi, 2009). From this view, concern for issues like war and climate change is not just a negative emotional response but also an expression of agency, where individuals represent themselves as active participants in a network of social and environmental interdependence. This awareness helps build shared meanings that go beyond individualism, rooted in an interconnected view of the world (Tajfel & Turner, 2004). These meanings foster prosocial commitments and actions aimed at the common good and personal wellbeing. Thus, investing in oneself and the community can be seen through the concept of *Eudaimonia*, a form of wellbeing that surpasses simple pleasure (i.e., Hedonia) and is based on personal fulfilment, meaning, purpose, and connection (Ryff, 1989; Deci & Ryan, 2000). Recent research shows that commitment to global causes, especially those emphasising justice, solidarity, and sustainability, relates to higher psychological wellbeing (Martela & Steger, 2016; Prilleltensky, 2012). Therefore, perceiving oneself as responsible for both personal and collective wellbeing helps create a stable and positive identity, reinforcing feelings of coherence, self-esteem, and belonging. Conversely, our results suggest that participants who are not concerned about war or climate change tend to hold meanings reflecting disconnection and disinvestment from themselves and their communities. This attitude may indicate alienation and resignation, which diminish a sense of agency and commitment to important goals. Without shared goals and meaningful connections, individuals risk experiencing a loss of purpose, isolation, and decreased psychological wellbeing. The study conducted by Randall (2009) posits that an asymmetry between the catastrophic impact of social phenomena and individual agency can give rise to a set of overwhelming emotions such as helplessness, loss, and apathy. While the evidence provided in Randall’s research is primarily focused on the effects of climate change, the model proposed by the authors appears to be applicable to other social phenomena, including warfare. Feelings of helplessness, loss, and apathy may lead to “uncomfortable knowledge” (Rayner, 2012), which in turn prompts individuals and groups to seek protection and consequently refuse responsibility. Based on scientific evidence, Lucas & Davison (2019) indicate that resistance to worry about large-scale phenomena—such as climate change in the context of their study—is also associated with political disengagement and diminished trust in government institutions. This attitude of distancing and disengagement corresponds with the description of our first latent dimension of meaning, namely the positive polarity. Furthermore, according to the author, feelings of distrust in social institutions, lack of self-efficacy, and fear can evoke fatalism. Fatalistic worldviews deny responsibility for social and collective issues by characterising them as unpredictable, uncontrollable, and resistant to human intervention. This underscores the significance of internal human dimensions and their potential to either facilitate or impede meaningful engagement. Wamsler et al. (2023) contend that negative emotions such as anxiety and frustration restrict individuals’ capacity to sustain transformative actions, whereas confidence in one’s agency coupled with positive emotions—such as hope and the sense of interconnectedness—enhances wellbeing and promotes active engagement.

The present research has limitations that should be acknowledged. First, data were collected online, and voluntary participants were mainly women in both the Armenian and Italian samples. This preponderance of females is typical in online surveys (Becker, 2022; Ramsey et al., 2016), and in our sample, it probably also reflects the greater interest of women in sensitive issues such as the environment and the global crises (Barchielli et al., 2022; Searle & Gow, 2010). Secondly, an online survey dissemination (i.e., via social networks) is a practical recruitment procedure, but it does not ensure the samples are representative. Some contextual factors, such as geographical location (e.g., rural/urban residence, proximity to conflict zones, or areas affected by natural disasters) or levels of stressor exposure (e.g., direct involvement and/or media exposure; Lau et al., 2024), could intervene by influencing emotional responses and psychological wellbeing of young people. Therefore, these factors may be deepened in future studies with larger national samples.

## 5. Conclusions

This study aimed to make a significant contribution to the psychological and contextualised understanding of youth wellbeing. Social phenomena, acting as potential stressors, can lead to highly differentiated subjective experiences. In this sense, wellbeing and distress do not follow a linear path. The findings support the idea that wellbeing is not simply the absence of disease, but a broader existential state, embedded in micro- and macro-social, historical, and cultural processes.

At a practical level, the findings from this study guide us toward increasingly personalised and context-sensitive interventions, recognising the individual in constant exchange with their social and community environments. These environments constitute spaces in which symbolic resources are strengthened and guide both thought and action, influencing the interpretation of experiences and wellbeing.

Based on the role played by meanings, the findings of this study invite us to analyse the impact of life events on youth health, shifting our focus from simply observing events to understanding how individuals attribute meaning to these experiences. The event itself does not mediate the effects of an event, but by the personal meanings inherent in the individual’s subjective narrative.

Overall, these perspectives support the adoption of systemic approaches that integrate both the complexity of social contexts and the semiotic processes underlying individual wellbeing, thus promoting more holistic and effective models of health psychology.

## Figures and Tables

**Table 1:** Cluster of meanings and cross-cultural differences for all measures (ANOVA).

Variable	Mean (SD [SE])						F_Cluster_ (*p*)	F_Group_ (*p*)	F_Cluster*Group_ (*p*)
	CL_1_		CL_2_		CL_3_				
	Armenia M (SD) [SE]	Italy M (SD) [SE]	Armenia M (SD) [SE]	Italy M (SD) [SE]	Armenia M (SD) [SE]	Italy M (SD) [SE]			
MHC-SF	30.3 (16.3) [4.71]	36.1 (18.8) [4.44]	33.1 (10.5) [0.99]	35.9 (14.3) [1.07]	31.7 (10.8) [1.22]	36.1 (15.3) [1.78]	0.197 (0.821)	5.327 (0.021)	0.264 (0.768)
DASS-21	42.1 (27.0) [7.80]	41.8 (26.3) [6.20]	48.5 (29.4) [2.78]	47.6 (29.6) [2.22]	41.3 (22.9) [2.60]	50.1 (30.1) [3.50]	0.795 (0.452)	0.395 (0.530)	1.456 (0.234)
IUS-R	32.4 (9.4) [3.65]	35.5 (9.2) [3.19]	34.6 (10.4) [0.99]	36.8 (9.5) [0.71]	28.0 (12.6) [1.07]	29.1 (13.5) [1.07]	7.284 (<0.001)	2.348 (0.126)	0.186 (0.830)
FTP	4.9 (0.7) [0.33]	4.6 (1.2) [0.24]	5.1 (0.8) [0.08]	4.9 (1.0) [0.08]	4.9 (1.2) [0.07]	4.1 (1.0) [0.14]	4.292 (0.014)	10.780 (0.001)	0.902 (0.407)
CCWS	18.4 (7.3) [2.83]	25.1 (9.0) [2.18]	21.6 (8.4) [0.79]	26.9 (9.7) [0.73]	19.1 (9.8) [0.83]	19.6 (9.2) [1.05]	6.543 (0.002)	10.790 (0.001)	1.532 (0.217)
WEWS	31.1 (7.8) [0.88]	27.2 (8.4) [0.97]	36.3 (9.8) [0.92]	29.8 (8.9) [0.66]	26.4 (11.0) [0.88]	20.4 (8.6) [0.97]	21.036 (<0.001)	18.791 (<0.001)	1.055 (0.349)

*Abbreviations*: CL_1_ = Orientation towards self-care; CL_2_ = Social and personal commitment; CL_3_ = Absolute devaluation and social detachment; M = Mean; SD = Standard Deviations; SE = Standard Error; F = Fisher test; *p* = Level of significance (*p*-value). *Measures*: MHC-SF = Mental Health Continuum-Short Form; DASS-21 = Depression Anxiety Stress Scale-21; IUS-R = Intolerance of Uncertainty Scale-Revised; FTP = Future Time Perspective Scale; CCWS = Climate Change Worry Scale; WEWS = War Experience Worry Scale. *Note*: Degree of freedom for clusters of meanings = 2, 467; Degree of freedom for nationality = 1, 467.

**Table 2:** Diagnostic prevalence for wellbeing disaggregated by (**a**) clusters of meanings, and (**b**) nationality.

**(a) Cluster of Meanings**	**Frequency of Continuum of Wellbeing from MHC (%)**			**Total** **n (%)**	**χ^2^ (df)**	***p***
	**Flourishing** **n (%)**	**Moderate** **n (%)**	**Languishing** **n (%)**			
CL_1_	26 (5.5)	103 (21.8)	23 (4.9)	152 (32.1)	12.024 (4)	0.017
CL_2_	51 (10.8)	195 (41.2)	45 (9.5)	291 (61.5)		
Cl_3_	9 (1.9)	11 (2.3)	10 (2.1)	30 (6.3)		
Total	86 (18.2)	309 (65.3)	78 (16.5)	473 (100.0)		
**(b) Nationality**	**Flourishing** **n (%)**	**Moderate** **n (%)**	**Languishing** **n (%)**	**Total** **n (%)**	**χ^2^ (df)**	***p***
Armenia	31 (6.6)	135 (28.5)	36 (7.6)	202 (42.7)	2.060 (2)	0.357
Italy	55 (11.6)	174 (36.8)	42 (8.9)	271 (57.3)		
Total	86 (18.2)	309 (65.3)	78 (16.5)	473 (100)		

*Abbreviations*: CL_1_ = Orientation towards self-care; CL_2_ = Social and personal commitment; CL_3_ = Absolute devaluation and social detachment; χ^2^ = Chi-square test; df = degree of freedom; *p* = Level of significance (*p*-value).

**Table 3:** Regression coefficients of the predictor variables and relative statistics on Mental Health Continuum (MHC-SF).

Predictors	Esteem	SE	t	*p*
**IUS-R**	**−0.236**	**0.0598**	**−3.94**	**<0.001**
**FTP**	**4706**	**0.6363**	**7.40**	**<0.001**
CCWS	0.112	0.0665	1.69	0.093
**WEWS**	**−0.194**	**0.0638**	**−3.04**	**0.003**
**VoC_1_**	**−3.484**	**13.364**	**−2.61**	**0.009**
VoC_2_	1.700	14.668	1.16	0.247

*Note*: *p* < 0.05 is considered statistically significant and marked in bold. IUS-R = Intolerance of Uncertainty Scale—Revised; FTP = Future Time Perspective scale; CCWS = Climate Change Worry Scale; VoC_1_ = First Dimension of Sense (“Levels of action in the social context”); VoC_2_ = Second Dimension of Sense (“Relationship with the social context”).

**Table 4:** Regression coefficients of the predictor variables and relative statistics on Distress (DASS-21).

Predictors	Esteem	SE	t	*p*
**IUS-R**	**13.072**	**0.111**	**11.727**	**<0.001**
**FTP**	**−76.063**	**1.186**	**−6.415**	**<0.001**
CCWS	0.0403	0.124	0.325	0.746
**WEWS**	**0.3421**	**0.119**	**2.876**	**0.004**
VoC_1_	23.631	2.490	0.949	0.343
VoC_2_	−37.617	2.733	−1.376	0.169

*Note*: *p* < 0.05 is considered statistically significant and marked in bold. IUS-R = Intolerance of Uncertainty Scale—Revised; FTP = Future Time Perspective scale; CCWS = Climate Change Worry Scale; VoC_1_ = First Dimension of Sense (“Levels of action in the social context”); VoC_2_ = Second Dimension of Sense (“Relationship with the social context”).

**Table 5:** Mediation analysis metrics (Predictor = CCWS; Mediator = VoC; Output Variable = MHC-SF).

Effect		Esteem	SE	Z	*p*	95% CI	
						Lower	Upper
Direct	CCWS > MHC-SF	0.046	0.063	0.737	0.461	−0.077	0.170
Indirect	**CCWS > VoC_1_ > MHC-SF**	**0.039**	**0.019**	**2.094**	**0.036**	**0.003**	**0.076**
	CCWS > VoC_2_ > MHC-SF	0.002	0.005	0.397	0.692	−0.007	0.011
Total	CCWS > MHC-SF	0.088	0.061	1.437	0.151	−0.032	0.207
Path coefficients	**CCWS > VoC_1_**	**−0.013**	**0.002**	**−6.372**	**<0.001**	**−0.017**	**−0.009**
	CCWS > VoC_2_	0.000	0.002	0.409	0.683	−0.003	0.005
	**VoC_1_ > MHC-SF**	**−2.943**	**1.328**	**−2.217**	**0.027**	**−5.545**	**−0.341**
	VoC_2_ > MHC-SF	2.406	1.452	1.657	0.097	−0.439	5.251
	**IUS-R > CCWS**	**0.194**	**0.043**	**4.470**	**<0.001**	**0.109**	**0.279**
	**FTP > CCWS**	**−1.187**	**0.450**	**−2.637**	**0.008**	**−2.069**	**−0.305**
	**IUS-R > VoC_1_**	**−0.009**	**0.002**	**−4.652**	**<0.001**	**−0.013**	**−0.005**
	**FTP > VoC_1_**	**−0.162**	**0.021**	**−7.847**	**<0.001**	**−0.203**	**−0.122**
	IUS-R > VoC_2_	−0.002	0.002	−0.862	0.388	−0.005	0.002
	FTP > VoC_2_	−0.038	0.019	−2.011	0.044	−0.075	0.000
	**IUS > MHC-SF**	**−0.254**	**0.060**	**−4.262**	**<0.001**	**−0.371**	**−0.137**
	**FTP > MHC-SF**	**4.639**	**0.637**	**7.278**	**<0.001**	**3.390**	**5.888**

*Note*: *p* < 0.05 is considered statistically significant and marked in bold. Grey area represents background variables. CCWS = Climate Change Worry Scale; MHC-SF = Mental Health Continuum—Short Form; FTP = Future Time perspective scale; IUS-R = Intolerance Uncertainty Scale—Revised; VoC_1_ = First Dimension of Sense (“Levels of action in the social context”); VoC_2_ = Second Dimension of Sense (“Relationship with the social context”).

**Table 6:** Mediation analysis metrics (Predictor = WEWS; Mediator = VoC; Output Variable = MHC-SF).

Effect		Esteem	SE	Z	*p*	95% CI	
						Lower	Upper
Direct	**WEWS > MHC-SF**	**−0.159**	**0.060**	**−2.642**	**0.008**	**−0.277**	**−0.041**
Indirect	**WEWS > VoC_1_ > MHC-SF**	**0.039**	**0.015**	**2.583**	**0.010**	**0.009**	**0.069**
	WEWS > VoC_2_ > MHC-SF	−0.010	0.009	−1.169	0.242	−0.027	0.007
Total	WEWS > MHC-SF	−0.130	0.059	−2.206	0.027	−0.245	−0.014
Path coefficients	**WEWS > VoC_1_**	**−0.010**	**0.002**	**−4.798**	**<0.001**	**−0.014**	**−0.006**
	**WEWS > VoC_2_**	**−0.005**	**0.002**	**−2.993**	**0.003**	**−0.009**	**−0.002**
	**VOC_1_ > MHC-SF**	**−3.979**	**1.298**	**−3.066**	**0.002**	**−6.523**	**−1.435**
	VoC_2_ > MHC-SF	1.851	1.458	1.270	0.204	−1.006	4.708
	**IUS-R > WEWS**	**0.201**	**0.045**	**4.468**	**<0.001**	**0.113**	**0.289**
	FTP > WEWS	0.493	0.466	1.059	0.290	−0.420	1.407
	**IUS-R > VoC_1_**	**−0.010**	**0.002**	**−4.867**	**<0.001**	**-0.014**	**−0.006**
	**FTP > VoC_1_**	**−0.142**	**0.021**	**−6.767**	**<0.001**	**-0.183**	**−0.101**
	IUS-R > VoC_2_	0.000	0.002	−0.185	0.853	−0.004	0.003
	FTP > VoC_2_	−0.036	0.019	−1.947	0.052	−0.073	0.000
	**IUS-R > MHC-SF**	**−0.227**	**0.059**	**−3.819**	**<0.001**	**-0.343**	**−0.110**
	**FTP > MHC-SH**	**4.489**	**0.620**	**7.235**	**<0.001**	**3.273**	**5.705**

*Note*: *p* < 0.05 is considered statistically significant and marked in bold. Grey area represents background variables. WEWS = War Experience Worry Scale; FTP = Future Time perspective scale; IUS-R = Intolerance Uncertainty Scale 12; MHC-SF = Mental Health Continuum—Short Form; VoC_1_ = First Dimension of Sense (“Levels of action in the social context”); VoC_2_ = Second Dimension of Sense (“Relationship with the social context”).

## Data Availability

The data are available upon motivated request from the corresponding authors: M.I. and S.R.

## Appendix A

### Appendix A.1. Participants and Recruitment

Participants were recruited through invitations and notices distributed via a snowball sampling method using social networks. Data collection took place from May to October 2024 in Armenia and from September 2023 to July 2024 in Italy. Inclusion criteria: being between 18 and 35 years of age and being able to understand the instructions and statements within the questionnaires. Table A1 outlines the demographic characteristics of the participants, disaggregated by nationality.

**Table A1:** Participants’ demographic characteristics disaggregated by nationality.

Demographic Characteristics		*N* (%)		χ^2^	*p*
		Italy	Armenia		
		271 (57.3)	202 (42.7)		
Gender	Male	77 (16.3)	51 (10.8)	0.588	0.443
	Female	194 (41.0)	151 (31.9)		
Educational status	Elementary	35 (7.4)	0 (0.0)	124.052	<0.001
	Middle	144 (30.4)	43 (9.1)		
	High	7 (1.5)	25 (5.3)		
	Bachelor’s degree	70 (14.8)	133 (28.1)		
	Master	15 (3.2)	1 (0.2)		
Social status	Single	0 (0.0)	1 (0.2)	21.002	<0.001
	Married	257 (54.3)	166 (35.1)		
	Divorced	14 (3.0)	32 (6.8)		
	Widower	0 (0.0)	3 (0.6)		

*Abbreviations*: *N* = Sample size; χ^2^ = Chi-square test; *p* = Level of statistical significance (*p*-value).

### Appendix A.2. Measures

All measures were combined into a comprehensive questionnaire, which was made accessible online via a link or QR code using Google Forms. After the informant’s consent, the questionnaire asked about demographic characteristics (i.e., gender, age, social status, and educational attainment). The instruments are:

The *Mental Health Continuum—Short Form* (MHC-SF; Keyes, 2018) assesses well-being and positive functioning through 14 items that reflect wellbeing symptoms experienced over the past month. Responses are on a Likert scale from 0 (“Never”) to 5 (“Every day”). It includes three subscales measuring different aspects of well-being: emotional (Hedonic; MHC-E; 3 items), social (Eudaimonic; MHC-S; 5 items), and psychological (Eudaimonic; MHC-P; 6 items). For all of these subdimensions, higher scores indicate better positive functioning. The MHC-SF has demonstrated cross-cultural validity and high internal reliability, with Armenian (Cronbach’s α = 0.81; Żemojtel-Piotrowska et al., 2018) and Italian populations (α = 0.86; Petrillo et al., 2015). In this study, internal consistency was good for both Armenian (α Emotional = 0.79; α Psychological = 0.80; α Social = 0.65; α Total = 0.86) and Italian participants (α Emotional = 0.79; α Psychological = 0.89; α Social = 0.86; α Total = 0.92). Furthermore, MCH-SF allows a categorical assessment of mental health status within three conditions (flourishing, i.e., high well-being levels; languishing, i.e., low well-being levels; moderate mental health, corresponding to an intermediate condition between the two previous extremes). As Keyes (2018) affirms, «a diagnosis of flourishing is made if someone feels 1 of the 3 hedonic well-being symptoms (items 1–3) “every day” or “almost every day” and feels 6 of the 11 positive functioning symptoms (items 4–14) “every day” or “almost every day” in the past month. Languishing is the diagnosis when someone feels 1 of the 3 hedonic well-being symptoms (items 1–3) “never” or “once or twice” and feels 6 of the 11 positive functioning symptoms (items 4–8 are indicators of Social well-being and 9–14 are indicators of Psychological well-being) “never” or “once or twice” in the past month. Individuals who are neither “languishing” nor “flourishing” are then coded as “moderately mentally healthy.”» (p. 7/8).

*Depression Anxiety Stress Scale-21* (DASS-21; Lovibond & Lovibond, 1995) is derived from the comprehensive 42-item version and evaluates the frequency of emotional states related to depression (e.g., dysphoria, lack of interest/involvement), anxiety (e.g., somatic and psychological automatic arousal), and stress (e.g., tension, over-reactivity) through three distinct 7-item scales. Participants respond to each item using a 4-point Likert scale, ranging from 0 (i.e., “Did not apply to me at all”) to 3 (i.e., “Applied to me very much, or most of the time”). Total sub-scores are obtained by summing the responses for each of the three domains—depression, anxiety, and stress—and multiplying them by 2. A total score may be calculated by summing the three sub-scores, serving as a measure of overall distress; higher scores indicate greater severity of distress (Bottesi et al., 2015). Cross-cultural research indicates the broad applicability of this instrument across various countries with healthy general populations (Lee et al., 2019). In the present study, the Armenian and Italian samples completed the versions of the DASS-21 adapted to their respective national contexts, as developed by Erofeeva et al. (2021) and Zolotareva (2020), and by Bottesi et al. (2015), respectively. The internal consistency of the measures was found to be satisfactory in both Armenian (α Depression = 0.85; α Anxiety = 0.82; α Stress = 0.79; α Total = 0.93) and Italian samples (α Depression = 0.91; α Anxiety = 0.89; α Stress = 0.91; α Total = 0.95).

*The Intolerance of Uncertainty Scale-Revised* (IUS-R; Bottesi et al., 2019; Carleton et al., 2007) is a short 12-item questionnaire designed to assess the fear of the unknown across two dimensions, designated as “Prospective Anxiety” (i.e., the propensity of individuals to seek information to mitigate uncertainty actively; IUS-P; 7 items) and “Inhibitory Anxiety” (i.e., paralysis or avoidance responses in the face of uncertainty; IUS-I; 5 items). Items are rated on a 5-point Likert scale, ranging from 1 (i.e., “Strongly disagree”) to 5 (i.e., “Strongly agree”). The scale yields two separate scores and a total score (ranging from 12 to 60), obtained by aggregating the responses; higher scores indicate a greater intolerance of uncertainty. The Cronbach’s alphas for separate factors were good (all αs > 0.80) both for the Italian questionnaire (Bottesi et al., 2019) and those for the Armenian version were realized through a back-translation procedure for this study. Here total scores were used.

*The Future Time Perspective Scale* (FTP; Carstensen & Lang, 1996; Lang & Carstensen, 2002) is a 10-item questionnaire, in which respondents indicate their level of agreement with each statement using a 7-point Likert scale, ranging from 1 (i.e., “Very untrue”) to 7 (i.e., “Very true”). This scale evaluates perceptions of remaining opportunities in life (7 items) as well as remaining time in life (the last three items are reverse-scored). Scoring involves calculating the average response across all 10 items; higher scores suggest a longer time horizon. The original, updated scale and the Italian version and scoring instructions are accessible online through the Stanford Life-span Developmental Laboratory’s website (https://lifespan.stanford.edu/). The Armenian adaptation of the FTP was developed via a back-translation procedure for this study. Both the Armenian and Italian versions demonstrated satisfactory internal consistency, with Cronbach’s alpha coefficients of 0.62 and 0.85, respectively.

*The Climate Change Worry Scale* (CCWS; Stewart, 2021) is a 10-item scale designed to assess proximal concerns and perceived risks associated with climate change. An example item is “I worry that outbreaks of severe weather may be the result of a changing climate”. Responses are measured on a 5-point Likert scale indicating frequency, ranging from “Never” to “Always”. Higher scores denote more intense worry. The scale demonstrates excellent internal consistency, with Cronbach’s alpha values of 0.95 in the original version and 0.98 in the Italian adaptation, which consists of eight items (Innocenti et al., 2022). In the current study, the Italian 10-item instrument exhibited an excellent reliability coefficient (α = 0.93), and the Armenian translation, developed for this study, also demonstrated robust internal consistency (α = 0.89).

*The War Experience Worry Scale* (WEWS; 10 items) was developed for this study (Rollo et al., 2023). The WEWS items were generated by adapting Stewart’s (2021) CCWS statements to an armed conflict condition. A sample item is “I worry about how the effects of war might affect the lives of people I care about”; the response range was from 1 (i.e., “Never”) to 5 (i.e., “Always”), with higher scores corresponding to more intense negative thoughts about war and its consequences. The internal consistency of the scale was excellent in both the Armenian (α = 0.90) and Italian versions (α = 0.92).

The *Views of Context* (Ciavolino et al., 2017) is a self-report questionnaire designed to delineate the cultural context of a specific population and to identify the subjective meanings present within that cultural framework (Mossi & Salvatore, 2011; Venuleo et al., 2016). The cultural context is examined in terms of the set of meanings it provides to individuals for describing the social environment and experience. The *Views of Context* comprises 46 items designed to facilitate the expression of perceptions, opinions, and judgments regarding both the micro and macro social aspects (i.e., evaluation of the locality where the individual resides and the reliability of social facilities) as well as social identity (e.g., moral judgments concerning critical social behaviours). The items are associated with a 4-point Likert scale (ranging from “Little” to “A lot”). Examples of items include: “The people of your country are only interested in themselves and their families” and “People in life can only rely on themselves”. In this study, the *Views of Context* shows good internal consistency, as evidenced by Cronbach’s alpha coefficients from Armenian (α = 0.90) and Italian participants (α = 0.94).

### Appendix A.3. Multiple Correspondence Analysis

Multiple Correspondence Analysis (MCA) is a specific form of Principal Component Analysis for categorical variables (Greenacre & Blasius, 2006). It effectively synthesises and visualises the relationships among the variables, analysing a “subjects x variables” matrix with *I* rows (respondents) and *J* columns (response modality). The patterns of association among variables are summarised by a limited number of Factor Dimensions (Greenacre & Blasius, 2006), which explain a decreasing proportion of variability in response relationships across the sample. This indicates that a small number of factors capture most of the information in the data. Each factor dimension reflects opposition between two co-response patterns, interpreted as arising from a latent, generalised meaning linking response modalities beyond their specific content (Lebart et al., 1984). Consequently, the factors serve as indicators of an opposing dimension called the Latent Dimension of Sense (Mossi & Salvatore, 2011). The subjects’ scores (factor coordinates) on these dimensions measure their positioning relative to particular meanings. A respondent’s score on a dimension increases as their response profile aligns more closely with that dimension’s profile.

### Appendix A.4. Dimension of Meanings

The Benzécri formula for inertia adjustment (Benzécri, 1979) was used to evaluate the significance of the eigenvalues, and thus of the extracted factors. The Benzécri correction method is a statistical technique employed in multivariate data analysis, especially in correspondence analysis MCA. It sets a threshold value, equal to the ratio 1/*p*, where “*p*” is the number of active variables (i.e., 46 in this study), below which an eigenvalue and the associated factor are considered negligible. This allowed us to focus on the first two factor dimensions, called VoC_1_ and VoC_2_, derived from MCA. These dimensions account for most of the inertia—that is, total variance—in the data matrix (Abdi & Valentin, 2007). Specifically, VoC_1_ explains 54.1% of the inertia, and VoC_2_ explains 20.0%, together representing 74.1% of the total inertia. Table 2 and Table 3 display the most important response modalities that define the polarities of VoC_1_ and VoC_2_. On a geometric plane, the factor coordinate can range from negative to positive scores: the former placing the participant on the negative polarity of the factor dimension (negative numbers), and the latter on the positive polarity (positive numbers). On the first factor dimension, these correspond to left and right polarity, respectively; on the second dimension, down and up, respectively. For both dimensions, scores near zero suggest that the participants perceive themselves as positioned midway between the two polarities.

#### Appendix A.4.1. First Dimension (VoC_1_)

The first dimension of meanings has been interpreted as “Levels of action in the social context” due to its contrast between two polarities: “Commitment to oneself and others” (VoC_1_^NEG^) and “General disengagement” (VoC_1_^POS^).

The VoC_1_^NEG^ demonstrates a positive attitude characterised by extreme response tendencies, such as “very” and “very important”. It is significantly important to prioritise personal health, invest responsibly in one’s future through activities such as studying and reflecting on happiness, and to also care for the environment, uphold societal rules, respect others’ ideologies, and sustain family relationships. Furthermore, there is a prevalent concern for global issues, including warfare and climate change, which are believed to be largely caused by mentalities that lack proper education and regulatory frameworks. Individuals feel a strong obligation to care for themselves and their families.

Conversely, the VoC_1_^POS^ indicates a disregard for social values such as respecting rules, others’ ideologies, environmental stewardship, and personal values. This perspective suggests a diminished emphasis on investing in oneself through education, health, or happiness. Concern for global affairs is deemed unnecessary, and services like researchers, social media, newspapers, and television are viewed as unreliable sources of information.

In summary, a prevalent attitude of dedication to oneself and others, concerning the acknowledgement of the significance of rules of coexistence and collective values, is contrasted by a position of detachment and disinvestment, wherein values and harmony are considered superfluous (see Table A2).

**Table A2:** Response modes most significantly associated with the first factorial dimension (VoC_1_) “Levels of action in the social context”.

Test Value ^a^	Item	Modality of Response
Commitment to oneself and others (VoC_1_^NEG^)		
−13.87	Taking care of your health	Very important
−12.40	Investing in your future	Very important
−11.52	Thinking about your own happiness	Very important
−11.36	Taking care of the environment	Very important
−11.09	War is caused by people’s mentality	Strongly agree
−10.77	Studying	Very important
−10.75	War is caused by people’s selfishness	Strongly agree
−10.15	Respect the rules	Very important
−9.77	Respecting the ideologies of others	Very important
−9.76	Climate change is caused by people’s mentality	Strongly agree
−9.67	Staying with family	Very important
−9.59	Climate change is caused by lack of education	Strongly agree
−9.03	Climate change is caused by insufficient regulations	Strongly agree
−8.99	Worry about things that happen in the world	Very important
−8.93	I feel called to take care of myself	A lot
−8.71	War is caused by lack of education	Strongly agree
−8.40	I feel called to take care of my family	A lot
−8.39	Scholars are reliable	A lot
−6.75	I think I am responsible for my future	A lot
−6.65	Newspaper and TV are reliable	Quite
−6.16	I feel like I belong to the world	A lot
−5.68	I feel called to take care of the community	A lot
General disengagement (VoC_1_^POS^)		
12.56	Respect the rules	Not at all important
11.92	Taking care of the environment	Not at all important
11.61	Studying	Not at all important
11.40	Worry about things that happen in the world	Not at all important
11.01	Thinking about your own happiness	Not at all important
10.76	Investing in your future	Not at all important
10.69	Staying with family	Not at all important
10.59	Taking care of your health	Not at all important
9.91	Scholars are reliable	Not at all
9.66	Taking care of the environment	Slightly important
9.21	War is caused by people’s selfishness	Not at all
9.02	Respecting the ideologies of others	Slightly important
9.00	Climate change is caused by people’s mentality	Strongly disagree
9.00	War change is caused by people’s mentality	Strongly disagree
8.97	Climate change is caused by people’s selfishness	Strongly disagree
8.80	I think I am responsible for my future	Not at all
8.79	I feel called to take care of myself	Not at all
8.24	Social media are reliable	Not at all
8.16	I feel called to take care of my family	Not at all
8.07	I am satisfied with my health	Not at all
7.84	Newspaper and TV are reliable	Not at all
7.05	I feel like I belong to the world	Not at all

^a^ Coefficient of statistical association between an item and a factorial dimension.

#### Appendix A.4.2. Second Dimension (VoC_2_)

The second dimension of sense has been interpreted as “Relationship with the social context” because it contrasts two polarities: “Detachment and disillusionment” (VoC_2_^NEG^) and “Impotence and loneliness” (VoC_2_^POS^).

The VoC_2_^NEG^ indicates that a lack of education is recognised as a contributing factor to both war and climate change. War is depicted as a phenomenon related to individuals’ mentality, specifically their selfishness, as well as the presence of insufficient or inadequate regulations. A context where coexistence values are absent is acknowledged, such that respecting others’ ideologies is not considered important. This environment lacks prospects for improving living conditions. From this critical standpoint, from which individuals feel distanced (not identifying with their nation), a sense of ambivalence regarding the future emerges; consequently, studying and investing in one’s future are perceived as both highly important and not important at all, reflecting two opposing responses. Correspondingly, extreme and contradictory response modes coexist.

The VoC_2_^POS^ suggests that, on one hand, climate change and war are believed to stem from a lack of education and selfishness; on the other hand, there is an awareness of the importance of caring for oneself and the environment, and concern about global affairs. Additionally, these phenomena are sometimes attributed to divine will. Values such as coexistence (respect for others’ rules and ideologies, family cohesion) and self-care (pursuit of personal happiness, investing in one’s future, studying) are acknowledged. Nonetheless, the context is characterised by individuals who feel limited in their capacity to effect change, relying solely on themselves, which fosters a sense of personal responsibility for the future. Despite this, a sense of belonging to one’s nation is maintained. Overall, responses tend to be moderate on the Likert scale, indicating a balanced attitude.

Generally, it can be inferred that a lack of education is perceived as a root cause of global conflicts and climate change, with war viewed as a consequence of selfishness and inadequate social norms. In a setting marked by erosion of coexistence values and disillusionment regarding life improvement, ambivalence towards the future is evident, as reflected in conflicting attitudes towards studying and investing in one’s future. Conversely, climate change and war are viewed both as results of insufficient education, selfishness, and mentality, and as events connected to divine will. Consequently, an ambivalence exists regarding the extent of personal responsibility and the influence of external factors. While recognition of values related to coexistence, self-care, and the environment is apparent, the context suggests that individuals often feel isolated in their responsibility for the future. Nevertheless, a persistent sense of belonging to one’s nation remains (see Table A3).

**Table A3:** Response modes most significantly associated with the first factorial dimension (VoC_2_) “Relationship with the social context”.

Test Value ^a^	Item	Modality of Response
Detachment and disillusionment (VoC_2_^NEG^)		
−9.15	War is caused by lack of education	Strongly agree
−8.65	Climate change is caused by lack of education	Strongly agree
−8.00	War is caused by people’s selfishness	Strongly agree
−7.66	War is caused by insufficient or inadequate rules	Strongly agree
−7.61	Climate change is caused by people’s mentality	Strongly disagree
−7.56	War is caused by people’s mentality	Strongly agree
−7.54	Climate change is caused by insufficient or inadequate rules	Strongly disagree
−7.43	Respecting the ideologies of others	Not at all important
−7.40	Worry about things that happen in the world	Not at all important
−7.40	I think my living conditions will improve in the next few years	Strongly disagree
−7.19	I feel called to take care of myself	Not at all
−7.14	Taking care of the environment	Very important
−7.11	Climate change is caused by lack of education	Strongly disagree
−7.05	Studying	Not at all important
−6.97	Investing in your future	Not at all important
−6.95	I feel like I belong to my nation	Not at all
−6.85	Investing in your future	Very important
−6.83	I feel called to take care of my family	Not at all
−6.54	Respect the rules	Very important
−6.49	War is caused by people’s mentality	Strongly disagree
−6.49	Thinking about your own happiness	Very important
−6.48	Politicians are reliable	Not at all
Impotence and loneliness (VoC_2_^POS^)		
10.37	Climate change is caused by lack of education	Moderately agree
10.05	War is caused by people’s selfishness	Moderately agree
9.41	War is caused by lack of education	Moderately agree
9.15	Taking care of your health	Moderately important
8.78	Taking care of the environment	Moderately important
8.77	Studying	Moderately important
8.56	War is caused by people’s mentality	Moderately agree
8.49	Climate change is caused by people’s mentality	Moderately agree
8.43	Climate change is caused by people’s selfishness	Moderately agree
8.39	Thinking about your own happiness	Moderately important
7.82	Investing in your future	Moderately important
7.75	War is caused by insufficient or inadequate rules	Moderately agree
7.59	Respect the rules	Moderately important
7.49	Respecting the ideologies of others	Moderately important
7.36	Worry about things that happen in the world	Moderately important
7.06	I think I am responsible for my future	Moderately
6.91	War is caused by divine will	Moderately agree
6.25	Climate change is caused by insufficient or inadequate rules	Moderately agree
5.87	Climate change is caused by divine will	Moderately agree
5.44	I feel called to take care of myself	Moderately
5.35	People are not capable of change	A little
5.09	In life people can only rely on themselves	Moderately

^a^ Coefficient of statistical association between an item and a factorial dimension.

#### Appendix A.4.3. Cluster of Meanings

Three clusters (CL) were extracted. They are described below.

##### Orientation Towards Self-Care (CL_1_)

Individuals in CL_1_ (n_CL1_ = 152; Italian = 74; Armenian = 78; Mean Age = 20.8, SD = 3.0) acknowledge that global phenomena such as climate change and conflict are associated with selfish mentalities and deficiencies in education. Nevertheless, these events are also perceived as components of a divine plan. Valuations of coexistence and personal responsibility are affirmed, with a strong emphasis on self-care, scholarly pursuit, and investment in the future. In this context, the challenges associated with change are apparent; however, an awareness of personal responsibility for one’s future and a sense of national connection also emerge (see Table A4).

**Table A4:** First cluster of meanings (CL_1_) “Orientation toward self-care”.

Test Value ^a^	Item	Modality of Response
10.54	War is caused by people’s selfishness	Moderately agree
9.80	War is caused by people’s mentality	Moderately agree
9.32	Taking care of your health	Moderately important
8.70	Climate change is caused by lack of education	Moderately agree
8.56	Studying	Moderately important
8.49	Climate change is caused by people’s mentality	Moderately agree
8.31	Taking care of the environment	Moderately important
8.04	Investing in your future	Moderately important
7.82	Respect le rules	Moderately important
7.43	War is caused by lack of education	Moderately agree
7.30	Thinking about your own happiness	Moderately important
6.99	Respecting the ideologies of others	Moderately important
6.51	Climate change is caused by people’s selfishness	Moderately agree
5.69	Worry about things that happen in the world	Moderately important
5.63	Climate change is caused by insufficient regulations	Moderately agree
5.38	Climate change is caused by divine will	Moderately agree
4.99	Staying with family	Moderately important
4.71	War is caused by divine will	Moderately agree
4.51	I think I am responsible for my future	Moderately
4.49	Climate change is caused by insufficient regulations	Moderately disagree

^a^ Coefficient of statistical item aggregation.

##### Social and Personal Commitment (CL_2_)

Individuals classified as CL_2_ (n_CL2_ = 291; Italian = 179; Armenian = 112; Mean Age = 21.6, SD = 3.5) exhibit an attitude characterised by extreme responses. A pronounced sense of responsibility toward one’s health, future, personal happiness, and familial well-being is evident. Concurrently, the significance of environmental stewardship, adherence to rules, and respect for the ideologies of others is acknowledged. Nevertheless, there exists a critical awareness regarding global issues, such as war and climate change, which are viewed as outcomes of a self-centred mentality, insufficient education, and inadequate regulatory measures (see Table A5).

**Table A5:** Second cluster of meanings (CL_2_) “Social and personal commitment”.

Test Value ^a^	Item	Modality of Response
13.11	Climate change is caused by lack of education	Strongly agree
13.09	War is caused by people’s selfishness	Strongly agree
12.60	War is caused by people’s mentality	Strongly agree
11.80	Taking care of your health	Very important
11.64	Climate change is caused by people’s mentality	Strongly agree
11.61	War is caused by lack of education	Strongly agree
11.03	Taking care of the environment	Very important
10.84	Investing in your future	Very important
10.56	Studying	Very important
10.49	Climate change is caused by insufficient regulations	Strongly agree
10.46	Climate change is caused by people’s selfishness	Strongly agree
10.01	Respect the rules	Very important
9.63	Respecting the ideologies of others	Very important
9.29	War is caused by insufficient regulations	Strongly agree
9.28	Thinking about your own happiness	Very important
9.27	Worry about things that happen in the world	Very important
7.84	Scholars are reliable	A lot
7.82	Staying with family	Very important
6.78	I think I am responsible for my future	A lot
6.40	I feel called to take care of myself	A lot

^a^ Coefficient of statistical item aggregation.

##### Absolute Devaluation and Social Detachment (CL_3_)

Individuals belonging to CL_3_ (n_CL3_ = 30; Italian = 18; Armenian = 12; Mean Age = 20.3, SD = 3.1) exhibit a critical perspective towards contemporary society, wherein fundamental values such as respect for rules, consideration for others, environmental stewardship, and the appreciation of personal future prospects are deficient. The conviction that concerns over global events or personal well-being, such as studies or health, is unwarranted, reflects a context characterised by widespread indifference. In this context, the principles of coexistence and respect for ideological diversity are called into question, fostering a sense of disconnection from one’s immediate environment (see Table A6).

**Table A6:** Third cluster of meanings (CL_3_) “Absolute devaluation and social detachment”.

Test Value ^a^	Item	Modality of Response
9.39	Respect the rules	Not at all important
8.33	Taking care of your health	Slightly important
7.75	Studying	Not at all important
7.29	Studying	Slightly important
7.29	Taking care of the environment	Not at all important
7.21	Taking care of the environment	Slightly important
7.11	Worry about things that happen in the world	Not at all important
6.88	Thinking about your own happiness	Not at all important
6.80	Staying with family	Not at all important
6.67	Investing in your future	Not at all important
6.45	Respecting the ideologies of others	Not at all important
6.40	Scholars are reliable	Not at all
6.40	Thinking about your own happiness	Slightly important
6.37	Investing in your future	Slightly important
6.27	Taking care of your health	Not at all important
6.17	Respecting the ideologies of others	Slightly important
5.69	War is caused by people’s selfishness	Strongly disagree
5.59	Climate change is caused by people’s selfishness	Strongly disagree
5.56	War is caused by people’s mentality	Strongly disagree
5.24	Social media are reliable	Not at all

^a^ Coefficient of statistical item aggregation.

### Appendix A.5. Supplementary Tables for the Mediational Role of the Cultural Meanings

**Table A7:** Frequency of responses on Climate Change Worry Scale (CCWS) for overall sample and disaggregated for sites of recruitment (i.e., Armenia and Italy).

Item	Never/Rarely (%)			Sometimes (%)			Often/Always (%)		
	Overall	Armenia	Italy	Overall	Armenia	Italy	Overall	Armenia	Italy
1. I worry about climate change more than other people	52.9	63.3	45.0	29.4	23.8	33.6	17.8	12.9	21.4
2. Thoughts about climate change make me worry about what the future might hold	45.8	62.9	33.2	30.4	25.7	33.9	23.7	11.4	32.8
3. I tend to look for information about climate change in the media	60.3	70.3	52.8	22.6	16.3	27.3	17.1	13.4	19.9
4. I tend to get worried when I hear about climate change, even when the effects of climate change may be far away	55.0	68.8	44.7	22.6	21.3	23.6	22.4	9.9	31.7
5. I believe that the increase in severe weather events may be the result of climate change	44.8	66.3	28.8	17.5	15.3	19.2	37.6	18.3	52.0
6. I care so much about climate change that I feel paralyzed from being able to do anything about it	71.3	65.8	75.3	18.6	18.8	17.7	10.2	14.3	7.0
7. I fear I will not be able to cope with climate change	61.6	79.2	48.3	19.0	13.4	23.2	19.4	7.5	28.4
8. I realized that I was worried about climate change	54.4	68.8	43.5	23.7	20.3	26.2	22.0	10.9	30.3
9. When I start worrying about climate change, I find it hard to stop	76.3	85.2	69.7	15.6	10.9	19.2	8.1	4.0	11.1
10. I worry about how the effects of climate change may affect the lives of people I care about	48.4	60.4	39.5	25.4	21.8	28.0	26.2	17.9	32.5

**Table A8:** Frequency of responses on War Experience Worry Scale (WEWS) for overall sample and disaggregated for sites of recruitment (i.e., Armenia and Italy).

Item	Never/Rarely (%)			Sometimes (%)			Often/Always (%)		
	Overall	Armenia	Italy	Overall	Armenia	Italy	Overall	Armenia	Italy
1. I worry about war more than other people	32.5	24.2	38.7	36.4	37.6	35.4	31.1	38.1	25.9
2. Thoughts about war make me worry about what the future might hold	18.8	11.4	24.3	30.0	23.8	34.7	51.1	64.8	40.9
3. I tend to look for information about war in the media	35.3	33.7	36.5	27.5	22.3	31.4	37.2	44.0	32.1
4. I tend to get worried when I hear about war, even when the effects of war may be far away	20.9	12.4	27.3	26.0	22.8	28.4	53.1	64.8	44.2
5. I believe that the increase in severe discord events may be the result of war	19.9	15.8	22.9	25.8	21.8	28.8	54.3	62.4	48.3
6. I care so much about war that I feel paralyzed from being able to do anything about it	63.9	59.9	66.8	18.0	16.3	19.2	18.2	23.8	14.0
7. I fear I will not be able to cope with war	35.8	32.1	38.4	27.3	24.7	29.1	37.0	43.1	32.5
8. I realized that I was worried about war	24.7	15.8	31.3	31.1	25.2	35.4	44.1	58.9	33.2
9. When I start worrying about war, I find it hard to stop	56.1	45.1	64.1	23.9	27.7	21.0	20.0	27.3	14.8
10. I worry about how the effects of war may affect the lives of people I care about	22.2	7.9	32.8	22.8	18.8	25.8	55.0	73.2	41.3

**Table A9:** Mediation analysis metrics (Predictor = CCWS; Mediator = VoC; Output Variable = DASS-21).

Effect		Esteem	SE	Z	*p*	95% CI	
						Lower	Upper
Direct	CCWS > DASS-21	0.156	0.117	1.331	0.183	−0.074	0.386
Indirect	CCWS > VoC_1_ > DASS-21	−0.019	0.033	−0.568	0.570	−0.084	0.046
	CCWS > VoC_2_ > DASS-21	−0.004	0.010	−0.399	0.690	−0.023	0.015
Total	CCWS > DASS-21	0.133	0.113	1.180	0.238	−0.088	0.355

*Note*: CCWS = Climate Change Worry Scale; DASS = Depression, Anxiety, Stress Scale; VoC_1_ = First Dimension of Sense (“Levels of action in the social context”); VoC_2_ = Second Dimension of Sense (“Relationship with the social context”).

**Table A10:** Mediation analysis metrics (Predictor = WEWS; Mediator = VoC; Output Variable = DASS-21).

Effect		Esteem	SE	Z	*p*	95% CI	
						Lower	Upper
Direct	**WEWS > DASS-21**	**0.355**	**0.112**	**3.176**	**0.001**	**0.136**	**0.574**
Indirect	WEWS > VoC_1_ > DASS-21	−0.022	0.024	−0.891	0.373	−0.069	0.026
	WEWS > VoC_2_ > DASS-21	0.020	0.016	1.245	0.213	−0.012	0.052
Total	WEWS > DASS-21	0.353	0.108	3.266	0.001	0.141	0.565

*Note*: *p* < 0.05 are considered statistically significant and marked in bold. WEWS = War Experience Worry Scale; DASS = Depression, Anxiety, Stress Scale; VoC_1_ = First Dimension of Sense (“Levels of action in the social context”); VoC_2_ = Second Dimension of Sense (“Relationship with the social context”).
